# Genome Sequence of “*Anthococcus*,” a Novel Genus of the Family *Streptococcaceae* Isolated from Flowers

**DOI:** 10.1128/genomeA.01410-16

**Published:** 2016-12-15

**Authors:** Li-Oon Chuah, Kien-Pong Yap, Kwai Lin Thong, Min Tze Liong, Rosma Ahmad, Ahamed Kamal Shamila-Syuhada, Gulam Rusul

**Affiliations:** a Food Technology Division, School of Industrial Technology, Universiti Sains Malaysia, Minden, Penang, Malaysia; b Institute of Biological Sciences, Faculty of Science, University of Malaya, Kuala Lumpur, Malaysia; c Bioprocess Technology Division, School of Industrial Technology, Universiti Sains Malaysia, Minden, Penang, Malaysia

## Abstract

Here, we report the draft whole-genome sequence of “*Anthococcus*,” a novel genus of the family *Streptococcaceae* isolated from fresh flowers of a durian (*Durio zibethinus*) tree. The draft genome of *Anthococcus* sp. strain DF1 contains 2,157,756 bp, with a G+C content of 33.0%.

## GENOME ANNOUNCEMENT

Recently, several novel lactic acid bacterial (LAB) species from the genera *Fructobacillus*, *Lactobacillus*, and *Leuconostoc* have been successfully isolated from flowers cultivated in temperate regions (1–5). These studies have highlighted the importance of complex and microbial diversity in flowers, the understudied part of the plants (6, 7). “*Anthococcus*” represents a novel genus of *Streptococcaceae*, a family to other well-known genera, such as *Lactococcus* and *Streptococcus*, and also *Lactovum* (8). Based on physiological, biochemical, phenotypical, and phylogenetic studies (our unpublished data), *Anthococcus* can be classified into a novel genus of *Streptococcaceae*. The availability of this novel *Anthococcus* genome sequence will enrich/enhance our understanding on the microbiome of plants, particularly the flowers, and serve as a framework for future taxonomic and functional studies. In the present study, strain DF1 was earlier isolated from fresh durian flowers and phenotypically (API 50 CHL, API Strep, and API zym) and molecularly characterized (by random amplified polymorphic DNA-PCR [RAPD-PCR]). DF1 was first grown in all-purpose Tween (APT) broth (BD, Franklin Lakes, NJ, USA) supplemented with 1% (wt/vol) sodium pyruvate at 30°C for 24 to 48 h, and genomic DNA extraction of the overnight cultures was performed using the DNeasy blood and tissue kit (Qiagen, Germantown, MD, USA). The DNA sequencing was performed using Ilumina MiSeq and generated ~1.39 Gb reads of data with a 571× depth coverage and a 250-bp read length. Sequence trimming, quality control (QC), and *de novo* assembly were performed using CLC Genomics Workbench (CLC bio, Aarhus, Denmark), as previously described (9, 10). The assembled contigs were subsequently annotated with Rapid Annotations Using Subsystems Technology (RAST) (9) and curated as previously described (10, 11).

The genome size of strain DF1 is 2,157,756 bp (26 contigs; *N*_50_, 241,922 bp; longest contig size, 393,964 bp; mean sequence size, 82,991 bp) with a G+C content of 33.0%. The DF1 genome carries 2,250 coding sequences with six rRNA, 54 tRNA, and one transfer-messenger (tmRNA) gene. As in other LAB species (12), DF1 contains an elaborated clustered regularly interspaced short palindromic repeat (CRISPR) system (*cas1*, *cas2*, and one *csn1* and *csn2* family gene), which confers the strain with adaptive immunity against invading genetic elements. Interestingly, we also identified an array of genes that code for fibronectin/fibrinogen-binding protein, lyzozyme M1, a regulator of exopolysaccharide synthesis/biofilm formation, and cell wall surface anchor family protein, which potentially is beneficial to the survival of the bacterium in the host (5, 13). To note, a large number of coding sequences (CDSs) predicted in the genome of DF1 are hypothetical with unknown functions. This offers a great opportunity for the discovery of novel genes and their associated products that are of practical and commercial use. Besides, future comparative genomic study will deepen our understanding in respect to the taxonomic classification of *Streptococcaceae* and *Anthococcus*.

### Accession number(s).

This whole-genome shotgun project has been deposited at DDBJ/ENA/GenBank under the accession no. MKIR00000000. The version described in this paper is the first version, MKIR01000000.
